# CD44v6 overexpression related to metastasis and poor prognosis of colorectal cancer: A meta-analysis

**DOI:** 10.18632/oncotarget.14163

**Published:** 2016-12-24

**Authors:** Ji-Lin Wang, Wen-Yu Su, Yan-Wei Lin, Hua Xiong, Ying-Xuan Chen, Jie Xu, Jing-Yuan Fang

**Affiliations:** ^1^ Division of Gastroenterology and Hepatology, Key Laboratory of Gastroenterology and Hepatology, Ministry of Health, State Key Laboratory for Oncogenes and Related Genes, Renji Hospital, School of Medicine, Shanghai Jiao Tong University, Shanghai Institute of Digestive Disease, Shanghai 200001, China; ^2^ Department of Rheumatology, Renji Hospital, School of Medicine, Shanghai Jiao Tong University, Shanghai 200001, China

**Keywords:** CD44v6, prognosis, colorectal cancer, meta-analysis

## Abstract

CD44v6 has recently been reported as a biomarker for colorectal cancer. However, the clinical and prognostic significance of CD44v6 in colorectal cancer remains controversial. Therefore, we performed a meta-analysis to clarify this issue. A comprehensive literature search was performed using Medline, Embase and Web of Science, and the statistical analysis was conducted using Stata software. A total of twenty-one studies including 3918 colorectal cancer cases were included. The pooled analysis showed that CD44v6 overexpression in colorectal cancer was an independent prognostic marker correlating with lower 5-year overall survival rate (OR=0.78, 95%CI =0.67-0.91, p=0.001). CD44v6 overexpression was also associated with more lymph node invasion (OR=1.48, 95%CI= 1.02-2.15, p=0.04), and advanced Dukes stage (OR=2.47, 95%CI= 1.29-4.73, p=0.01). In addition, while excluding Zolbec's study, CD44v6 overexpression was associated with distance metastasis (OR=1.65, 95%CI =1.13-2.40, p=0.01). Taken together, this meta-analysis suggested that CD44v6 is an efficient prognostic factor in colorectal cancer.

## INTRODUCTION

Although the treatments for colorectal cancer (CRC) have developed rapidly in recent years, CRC still remains one of the most commonly diagnosed cancers and the second leading cause of cancer-related death worldwide [1, 2]. CRC patients with early stage generally have an relatively good prognosis after curative resection, but the prognosis for patients with lymph node invasion or distant metastases remains poor. Therefore, it is imperative to searching more specific histopathological markers for CRC progression.

As a compelling stem cell marker, CD44 has been reported to play important roles in tumor initiation and metastasis [3]. CD44 is a glycosylated cell surface molecule which belongs to a family of hyaluronan binding proteins. It is encoded by a single gene containing 20 exons and is located on the short arm of chromosome 11 (11p13), and has many variant isoforms generated by alternative splicing of at least 10 variant exons. All isoforms contain a constant region comprising a large ectodomain, a transmembrane domain, and a cytoplasmic domain. These domains are encoded by the first five exons and the last five exons (16-20), accounting for the smallest but ubiquitously expressed isoform CD44. Close to the trans-membrane region, a variable part encoded by various combinations of exons 6-15 can be included, giving rise to CD44 variant isoforms. The CD44 family is important in a variety of physiological and pathological processes, including wound healing, inflammation and cancer biology.

Among the isoforms of CD44, CD44v6 has evoked special interest. CD44v6, like all other isoforms, contains a hyaluronan-binding site in its extracellular domain and serves as a major cell surface receptor for hyaluronan [4]. CD44v6 is more specifically expressed in cancer tissues, but CD44 has an ubiquitously expression pattern [5]. Therefore, CD44v6 has attracted more interest than CD44 in terms of tumor markers, diagnosis, and treatment. In fact, CD44V6 has been implicated in promoting cancer progression by regulating the extracellular matrix, suppressing tumor apoptosis and promoting cell motility [6]. Increased levels of CD44V6 has been found in various types of tumors and could serve as a prognostic marker in various solid tumors, including gastric cancer [7], osteosarcoma [8], lung cancer [9], esophageal cancer [10], and hepatocellular cancer [11].

Many studies have been conducted to explore the relationship between CD44v6 and the prognosis of CRC [12–32]. Most of these researches suggested that overexpression of CD44V6 was associated with tumor metastasis and worse overall survival in patients with CRC. However, controversial results have been reported by some other studies. To further clarify these issues, we conduct this meta-analysis including all of the evidence to date.

## RESULTS

### Search results and study characteristics

**Figure 1:**
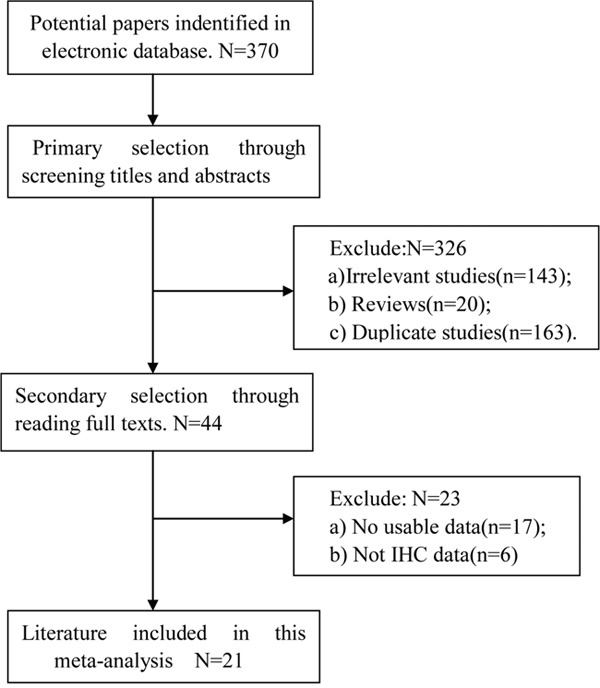
Flow chart for study selection

Three hundred and seventy potential papers were identified initially using the search strategy above, 326 of which were excluded after reading the titles and abstracts. After reading full texts, we excluded another 23 studies due to no usable data and not-immunohistochemistry method. Finally, 21 studies met the inclusion criteria and included in this meta-analysis, including 3918 cases. The detailed literature selection procedure was described in Figure 1. All these studies evaluated the expression of CD44v6 and risk of CRC by IHC staining method. The detailed characteristics of the studies are shown in Table 1.

**Table 1:** Characteristics of the included studies

Author [Ref]	Year	Country							N	Distance	TNM		5-year	5-year
			Number of cases	Duration follow-up	Tumor size (<5 cm/≥5 cm)	Tumor grade (G1+G2/G3)	Differentiation (Well/Poor)	T (T1,2/T3,4)	(Negative/Positive)	Metastasis (M0/M1)	(I+II/III+IV)	Dukes (A,B/C,D)	OS rate	DFS rate
Nihei [12]	###	Japan	42	5.9-71.3m	NA	NA	NA	NA	NA	H(8/13)	NA	H(9/12)	H(7/21)	NA
										L(2/19)		L(13/9)	L(18/21)	
Wielenga [13]	###	Netherland	68	6.5-9.5y	NA	NA	NA	NA	NA	NA	NA	NA	H(1/11)	NA
													L(13/13)	
Ropponen [14]	###	Finland	194	14y	NA	H(119/24)	NA	NA	NA	NA	NA	H(74/68)	H(53/133)	NA
						L(49/2)						L(49/2)	L(30/47)	
Yamane [15]	###	Japan	44	NA	NA	NA	H(10/7)	H(2/17)	H(8/11)	H(14/5)	NA	H(6/13)	H(8/19)	NA
							L(14/10)	L(5/20)	L(17/8)	L(23/2)		L(16/9)	L(2/25)	
Gu [16]	###	China	32	NA	NA	NA	NA	NA	H(10/9)	H(5/7)	NA	NA	NA	NA
									L(12/1)	L(13/0)				
Ishida [17]	###	Japan	63	NA	NA	NA	H(22/2)	H(10/14)	H(13/11)	H(19/5)	NA	H(14/11)	NA	NA
							L(34/6)	L(5/34)	L(20/19)	L(34/5)		L(20/19)		
Martin [18]	###	Germany	145	NA	NA	H(52/10)	NA	H(14/48)	H(36/26)	H(54/8)	NA	H(35/27)	H(41/62)	NA
						L(70/13)		L(28/55)	L(51/32)	L(78/5)		L(49/34)	L(60/83)	
Liu [19]	###	China	62	NA	NA	H(11/11)	NA	NA	H(10/12)	H(12/10)	NA	NA	NA	NA
						L(13/5)			L(13/5)	L(14/4)				
Liu [20]	###	China	50	NA	NA	NA	H(22/9)	NA	NA	H(23/9)	NA	H(9/23)	NA	NA
							L(14/5)			L(15/3)		L(13/5)		
Peng [21]	###	China	259	44m	NA	NA	NA	H(46/83)	H(70/59)	H(107/22)	H(70/59)	NA	H(107/129)	NA
								L(55/75)	L(77/53)	L(117/13)	L(77/53)		L(119/130)	
Peng [22]	###	China	179	3y	NA	NA	NA	NA	NA	NA	NA		H(39/75)	H(38/75)
													L(87/104)	L(84/104)
Zlobec [23]	###	Switerland	1279	NA	NA	H(770/113)	NA	H(177/703)	H(478/391)	H(243/42)	NA	NA	H(157/285)	NA
						L(306/37)		L(49/296)	L(159/178)	L(87/26)			L(54/112)	
Avoranta [24]	###	Finland	214	NA	NA	NA	NA	H(43/62)	H(68/37)	NA	NA	NA	NA	H(76/105)
								L(21/51)	L(40/32)					L(40/72)
Rao [25]	###	China	190	NA	NA	H(103/34)	NA	H(16/121)	H(81/56)	H(113/25)	H(69/68)	NA	H(29/58)	NA
						L(35/18)		L(3/50)	L(25/28)	L(42/10)	L(24/29)		L(41/53)	
Garouniatis [26]	###	Greece	183	72m	NA	NA	NA	NA	NA	NA	NA	NA	H(37/76)	NA
													L(90/107)	
Li [27]	###	China	57	5y	NA	NA	H(25/11)	NA	H(10/26)	NA	NA	H(8/28)	H(19/36)	NA
							L(20/1)		L(16/5)			L(16/5)	L(17/21)	
Saito [28]	###	Japan	133	NA	H(16/22)	NA	H(18/20)	H(1/37)	H(21/17)	NA	NA	NA	H(20/38)	H(21/38)
					L(38/37)		L(50/25)	L(3/72)	L(47/28)				L(65/75)	L(59/75)
Lee [29]	###	Korea	167	54.5m	NA	NA	NA	NA	NA	NA	NA	NA	H(25/46)	NA
													L(80/118)	
Wang [30]	###	China	203	NA	NA	NA	NA	NA	H(78/26)	NA	NA	NA	NA	NA
									L(86/13)					
Chen [31]	###	China	167	64m	H(45/55)	NA	H(4/91)	H(16/85)	H(93/8)	NA	H(16/85)	NA	H(87/101)	NA
					L(30/36)		L(2/62)	L(11/55)	L(64/2)		L(11/55)		L(48/66)	
Zhao [32]	###	China	187	NA	H(91/44)	NA	H(86/48)	H(24/101)	H(82/53)	NA	NA	NA	NA	NA
					L(42/10)		L(43/10)	L(15/74)	L(42/10)					

### Correlation of CD44v6 with clinicopathological features of CRC

**Figure 2:**
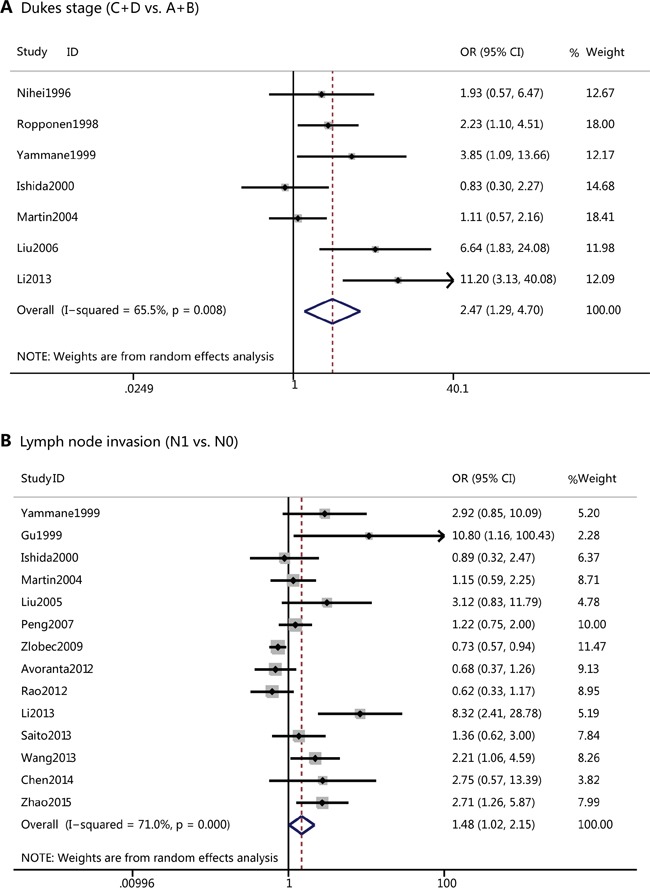
**A.**Forest plots of CD44v6 overexpression and Dukes stage of colorectal cancer; **B**. Forest plots of CD44v6 overexpression and lymph node invasion.

The association between CD44v6 and several clinicopathological features of CRC was illustrated in Table 2. The results of this meta-analysis showed CD44v6 overexpression was associated with lymph node invasion ((OR=1.48(positive versus negative), 95%CI= 1.02-2.15, p=0.04), and Dukes stage ((OR=2.47(Dukes C+D versus A+B), 95%CI= 1.29-4.73, p=0.01) (Figure 2). However, CD44v6 was not associated with other clinicopathological features such as T categary (OR=0.90, 95%CI =0.62-1.29, p=0.56), distance metastasis (OR=1.46, 95%CI =0.81-2.64, p= 0.21), tumor size (OR=1.37, 95%CI=0.91-2.06, p=0.13), tumor grade (OR=1.17, 95%CI=0.86-1.58, p=0.32), and tumor differentiation (OR=1.56, 95%CI =0.96-2.56, p=0.08), though there was a tendency that overexpression of CD44v6 related to more metastasis, larger tumor size and poor differentiation.

**Table 2:** CD44v6 with the clinicopathological features of colorectal cancer

Features	RR(95%CI)	P value	P_het_
T categary	0.90(0.62-1.29)	0.56	0.03
(T3+4/T1+2)			
Lymph node	1.48(1.02-2.15)	0.04	0.00
(N1/N0)			
Metastasis	1.46(0.81-2.64)	0.21	0.01
(M1/M0)			
Tumor size	1.37(0.91-2.06)	0.13	0.4
(Large/Small)			
Differentiation	1.56(0.96-2.56)	0.08	0.27
(Poor/Well)			
Grade	1.17(0.86-1.58)	0.32	0.08
(Grade3/Grade1+2)			
Dukes	2.47(1.29-4.70)	0.01	0.01
(Dukes C+D/A+B)			
5y-OS	0.78(0.67-0.91)	0.001	.09
DFS	0.85(0.63-1.15)	0.29	0.09

### Impact of CD44v6 on 5-year OS and DSF of CRC

**Figure 3:**
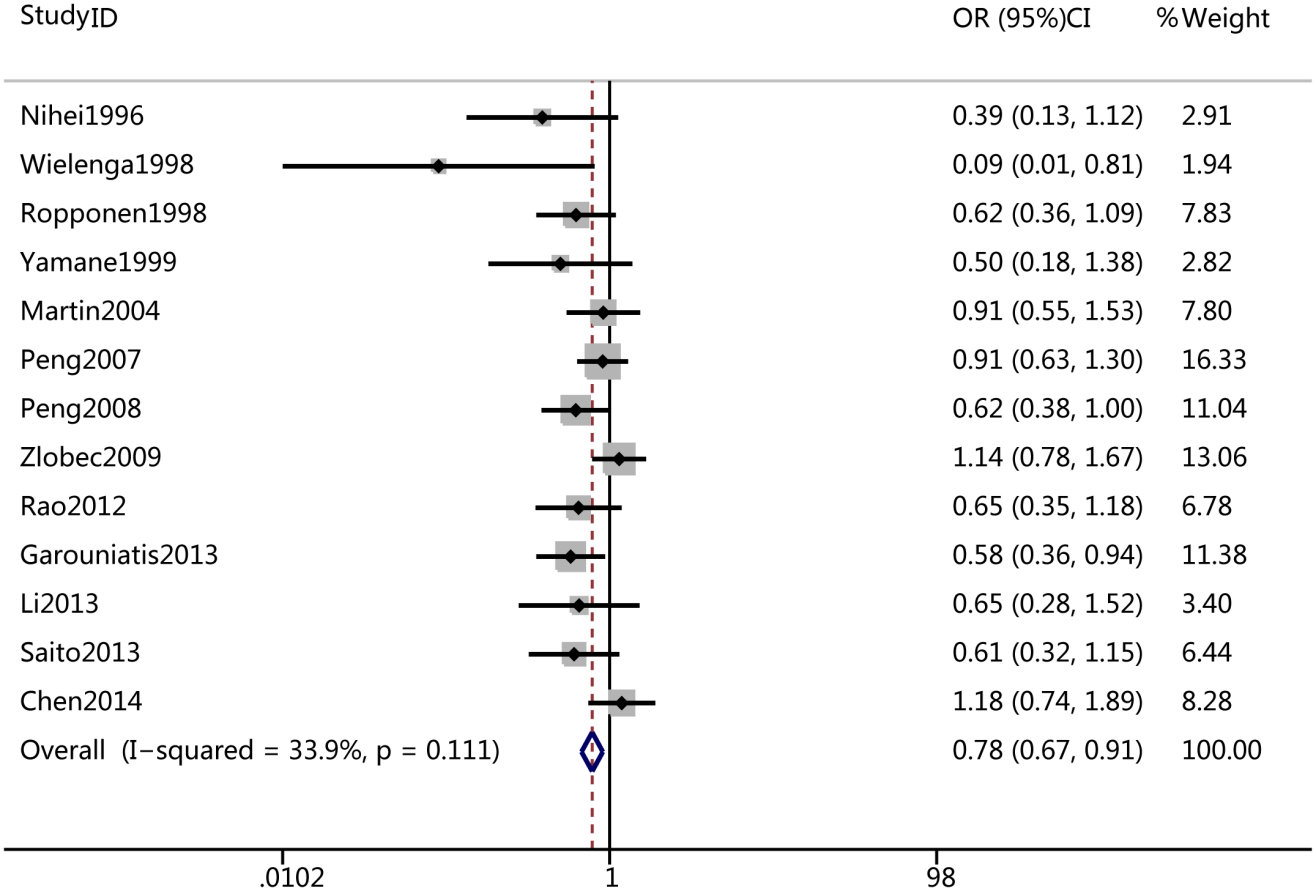
Forest plots of CD44v6 overexpression and 5-year overall survival rate

A total of thirteen studies reported the association of CD44v6 and 5-year OS rate of CRC, while only three studies reported the correlation of CD44v6 with 5-year DSF rate of CRC. The pooled analysis revealed that CD44v6 overexpression was significantly associated with poor 5-year OS rate in a fixed-effects model (OR=0.78, 95%CI =0.67-0.91, p=0.001) (Figure 3), 5-year OS rate was 0.78-fold lower in CD44v6-positive patients; There was also a tendency that overexpression of CD44v6 related to poor 5-year DFS rate, though the association was not statistically significant CRC (OR=0.85, 95%CI =0.63-1.15, p=0.09).

### Sensitivity analysis

**Figure 4:**
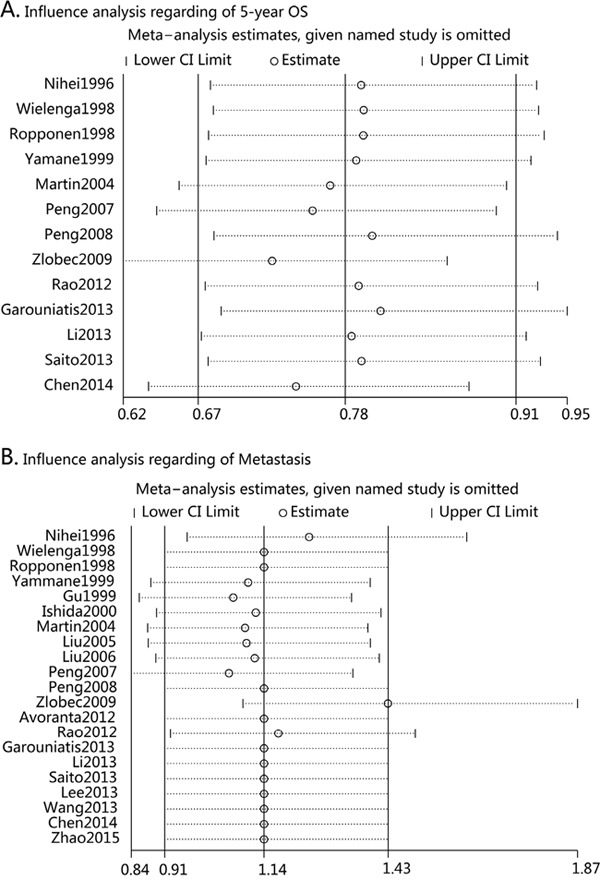
**A.** Influence analysis regarding 5-year overall survival rate; **B**. Influence analysis regarding Metastasis.

**Figure 5:**
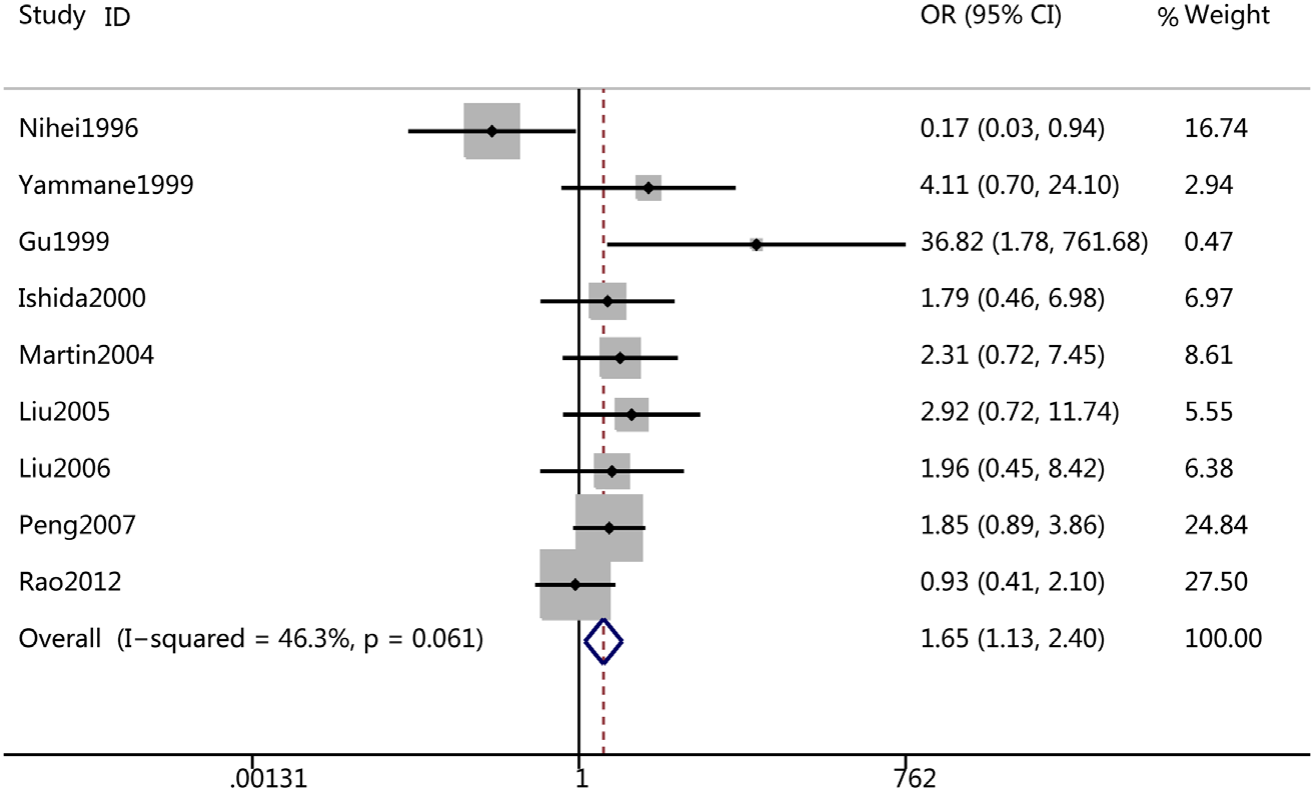
Forest plots of CD44v6 overexpression and metastasis after excluding Zlobec's study

Influence analysis was performed to assess the influence of each individual trial on the pooled ORs regarding of 5-year OS by sequential omission of individual trial. The analysis suggested that no individual trial could statistically significant affected the pooled ORs of 5-year OS (Figure 4A). However, we found that Zlobec's study may affect other pooled ORs such as that of metastasis (Figure 4B). In fact, while excluding Zlobec's study, the pooled analysis suggested that CD44v6 overexpression was associated with distance metastasis (OR=1.65, 95%CI=1.13-2.40, p=0.01) (Figure 5 and Table 3).

**Table 3:** CD44v6 and CRC after excluding Zlobec's study

Features	RR(95%CI)	P value	P_het_
T categary	1.01(0.77-132)	0.95	0.06
(T3+4/T1+2)			
Lymph node	1.62(1.09-2.40)	0.02	0.001
(N1/N0)			
Metastasis	1.65(1.13-2.40)	0.01	0.06
(M1/M0)			
Grade	1.24(0.46-3.36)	0.68	0.04
(Grade3/Grade1+2)			
5y-OS	0.72(0.62-0.86)	0.00	0.25

### Publication bias

**Figure 6:**
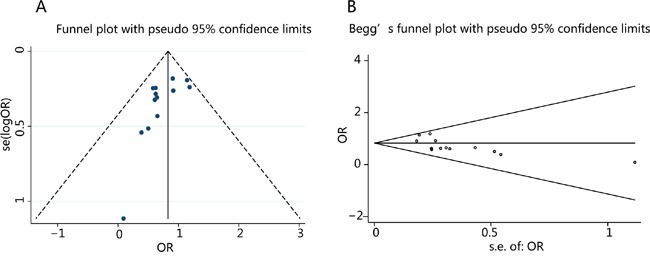
Funnel plot (A) and Begger's test (B) for publication bias

Potential publication bias was examined by funnel plots in the analysis of CD44v6 expression and 5-year OS rate. The shapes of the funnel plots was symmetric, suggesting no obvious publication bias. Furthermore, the p values assessed by the Begg's test and Egger's test were all greater than 0.05, indicating no obvious publication bias among these studies regarding the OR for 5-year OS rate (Figure 6).

## DISCUSSION

Cancer-stem cells (CSCs) are regarded to be significantly responsible for initiation, growth, metastasis and recurrence of various tumors [33]. Many cell surface molecular have been used to mark CSCs from different tumors, such as CD44, CD24, CD133, and aldehyde dehydrogenase1 (ALDH1) [34]. Regarding the biological properties of CSCs, many studies suggested that evaluation of CD44, specially its variant isoform CD44v6 expression in CRC tissues may be useful in the future as a novel prognostic factor. Despite a variety of clinical studies on CD44v6 and CRC, no consensus has been reached in detail. Based on the previous studies, we performed this meta-analysis to systematically review the correlation between overexpression and clinical significance of CD44v6 in CRC.

To our knowledge, this is the first meta-analysis which systematically evaluated the association between CD44v6 expression and the risk of CRC and its clinicopathological parameters. Our meta-analysis included 21 studies with a total of 3918 cases, reflecting a large sample size and strong statistical power. In this meta-analysis, we found that overexpression of CD44v6 is related to lymph node metastasis, Dukes stage of CRC, and distance metastasis. Furthermore, CD44v6 overexpressed CRC patients was of lower 5-year overall survival in comparison with negative cases. In conclusion, overexpression of CD44v6 and its clinicalpathological features are closely related in CRC. Our results provide further support to the potential translational significance of CD44v6 in CRC. Todaro and colleagues [35] found that CD44v6 expression is higher in metastasis CRC tissues and CD44v6-positive CRC stem cells are required for their migration and generation of metastatic tumors. Wang's study [36] suggested that CD44v6-competent tumor exosomes could promote motility and invasion by stimulating cancer-initiating cell marker expression. Matzke-Ogi found that peptide inhibitors of CD44v6 could block tumor growth and metastasis in several independent models of pancreatic cancer. This study also suggested that the CD44v6 peptide was more efficient than the MET inhibitor crizotinib and the VEGFR-2 inhibitor pazopanib in reducing xenograft tumor metastasis [37]. CD44v6 may also promote ovarian cancer cell invasion by promoting β-catenin and TGF-β expression [38]. Furthermore, overexpression of CD44v6 could contribute to chemoresistance in CRC cells under cytotoxic stress via the modulation of autophagy and EMT, and activation of the PI3K and MAPK pathways [39]. While our meta-analysis and many clinical studies have suggested oncogene properties of CD44v6, there are still some controversies between the overexpression and clinical significance of CD44v6 in CRC [23, 40, 41]. Therefore, more prospective study needs to be conducted on this issue.

Although this meta-analysis aimed to provide the best possible estimate of the correlation between the clinical significance of CD44v6 in CRC, it may have limitations. First, the number of included articles was relatively small with only about 3918 cases. Second, there was between-study heterogeneity in most of the analysis, this may be due to the different concentrations of antibodies, the different cut-off values of CD44v6 expression used in the studies. Third, although immunohistochemistry was the most commonly applied method for detecting CD44v6 in situ, RT-PCR method had also been used for the evaluation of the levels of CD44v6 mRNA expression in CRC tissue. However, these studies measuring CD133 mRNA level by RT-PCR were not yet included in this meta-analysis. Finally, publication bias may have occurred though the funnel plot did not show it since negative findings were likely to be unreported.

In summary, despite the limitations listed above, this meta-analysis shows a significant correlation between CD44v6 overexpression and 5-year OS rate as well as metastasis in CRC patients. CD44v6 may have prognostic significance for CRC patients based on currently obtained data. Further larger well-designed prospective studies are warranted to confirm these findings from our study.

## MATERIALS AND METHODS

### Search strategy

Eligible studies were retrieved by searching the following database: Medline, Embase, and Web of Science. The search strategy included the following keywords: “CD44V6”, “CD44 variation 6”, “colorectal cancer”, “colon cancer”, “rectal cancer”. In addition, the reference list of each primary study and of previous systematic reviews were also manually searched to avoid missing studies.

### Study selection

All eligible studies evaluated the association between CD44v6 expression and the risk of CRC were selected in this meta-analysis. Studies meeting the following inclusion criteria were included: 1) CD44v6 expression was detected in CRC by immunohistochemistry; 2) studies about the relationship between CD44v6 expression and the clinicopathological features or prognosis of CRC; 3) studies regarding the prognosis of CRC provided sufficient data to estimate hazard ratios (HRs) for overall survival (OS) or disease free survival (DFS). Those meta-analysis, reviews or systematic reviews, abstracts, letters and those provided not sufficient data and not using immunohistochemistry were exluded. We did not assess the methodologic quality of the included studies, given that quality scorings of observational studies in meta-analysis is controversial.

### Data extraction

All data were independently extracted by two investigators (JW and WS), and disagreements in data extraction were resolved by discussion. The following data were recorded from each article: the name of first author, publication year, country of the population studied, number of cases, duration of follow-up, T category, N category, distant metastasis, tumor size, histology, tumor grade, TNM stage, Dukes stage, and most importantly the 5-year OS rate and 5-year DFS rate. For those studies which didn't provide 5-year OS and DFS directly, Kaplan–Meier curves were read by GetData Graph Digitizer (http://getdata-graph-digitizer.com).

### Statistical analysis

Statistical analysis was performed using STATA 11.0 (Stata Corporation, College Station, Texas). Comparisons of CD44v6 expression in groups with different clinicopathologic features and prognosis of CRC were assessed by pooled estimates of risk ratio as well as the 95% CI. p values less than 0.05 were considered statistically significant. The chi-squared (χ2) test and I2 statistics was calculated to assess between-study heterogeneity. Fixed-effects models (Mantel-Haenszel) were used when there was no between-study heterogeneity, otherwise, the random effect models (DerSimonian and Laird) were used. A funnel plot was used for estimating the potential publication bias, while the degree of asymmetry was determined by Begg's and Egger's test. Influence analysis was conducted by omitting each study to find potential outliers. Two authors (JW and WS) performed the statistical analysis independently and got the same results.

## References

[1] Torre LA, Bray F, Siegel RL, Ferlay J, Lortet-Tieulent J, Jemal A (2015). Global cancer statistics, 2012. CA Cancer J Clin.

[2] Chen W, Zheng R, Zeng H, Zhang S, He J (2015). Annual report on status of cancer in China, 2011. Chin J Cancer Res.

[3] Zöller M (2011). CD44: can a cancer-initiating cell profit from an abundantly expressed molecule?. Nat Rev Cancer.

[4] Zlobec I, Günthert U, Tornillo L, Iezzi G, Baumhoer D, Terracciano L, Lugli A (2009). Systematic assessment of the prognostic impact of membranous CD44v6 protein expression in colorectal cancer. Histopathology.

[5] Heider KH, Kuthan H, Stehle G, Munzert G (2004). CD44v6: a target for antibody-based cancer therapy. Cancer Immunol Immunother.

[6] Jung T, Gross W, Zöller M (2011). CD44v6 coordinates tumor matrix-triggered motility and apoptosis resistance. J Biol Chem.

[7] Chen J, Li T, Liu Q, Jiao H, Yang W, Liu X, Huo Z (2014). Clinical and prognostic significance of HIF-1α, PTEN, CD44v6, and survivin for gastric cancer: a meta-analysis. PLoS One.

[8] Zhang Y, Ding C, Wang J, Sun G, Cao Y, Xu L, Zhou L, Chen X (2015). Prognostic significance of CD44V6 expression in osteosarcoma: a meta-analysis. J Orthop Surg Res.

[9] Jiang H, Zhao W, Shao W (2014). Prognostic value of CD44 and CD44v6 expression in patients with non-small cell lung cancer: meta-analysis. Tumour Biol.

[10] Hu B, Luo W, Hu RT, Zhou Y, Qin SY, Jiang HX (2015). Meta-Analysis of Prognostic and Clinical Significance of CD44v6 in Esophageal Cancer. Medicine (Baltimore).

[11] Fu Y, Geng Y, Yang N, Zhu N, Wang CZ, Su XC, Zhang HB (2015). CD44v6 expression is associated with a poor prognosis in Chinese hepatocellular carcinoma patients: A meta-analysis. Clin Res Hepatol Gastroenterol.

[12] Nihei Z, Ichikawa W, Kojima K, Togo S, Miyanaga T, Hirayama R, Mishima Y (1996). The positive relationship between the expression of CD44 variant 6 and prognosis in colorectal cancer. Surg Today.

[13] Wielenga VJ1, van der Voort R, Mulder JW, Kruyt PM, Weidema WF, Oosting J, Seldenrijk CA, van Krimpen C, Offerhaus GJ, Pals ST (1998). CD44 splice variants as prognostic markers in colorectal cancer. Scand J Gastroenterol.

[14] Ropponen KM, Eskelinen MJ, Lipponen PK, Alhava E, Kosma VM (1998). Expression of CD44 and variant proteins in human colorectal cancer and its relevance for prognosis. Scand J Gastroenterol.

[15] Yamane N, Tsujitani S, Makino M, Maeta M, Kaibara N (1999). Soluble CD44 variant 6 as a prognostic indicator in patients with colorectal cancer. Oncology.

[16] Gu J, Zhu X, Ye Y, Qu J, Huang L, Li R, Yu Y, Leng X (1999). The level of expression of adhesion molecules CD44v6 and E-cadherin in colorectal cancer and analysis of correlates with metastasis. Zhonghua Wai Ke Za Zhi.

[17] Ishida T (2000). Immunohistochemical expression of the CD44 variant 6 in colorectal adenocarcinoma. Surg Today.

[18] Köbel M, Weichert W, Crüwell K, Schmitt WD, Lautenschläger C, Hauptmann S (2004). Epithelial hyaluronic acid and CD44v6 are mutually involved in invasion of colorectal adenocarcinomas and linked to patient prognosis. Virchows Arch.

[19] Liu YJ, Yan PS, Li J, Jia JF (2005). Expression and significance of CD44s, CD44v6, and nm23 mRNA in human cancer. World J Gastroenterol.

[20] Lin HZ, Chen L, Zhou DF, Hao LH, Li XC, Chang H (2006). Study on the early liver metastasis forecast of colorectal neoplasms. Zhonghua Wai Ke Za Zhi.

[21] Peng JJ, Cai SJ, Lu HF, Cai GX, Lian P, Guan ZQ, Wang MH, Xu Y (2007). Predicting prognosis of rectal cancer patients with total mesorectal excision using molecular markers. World J Gastroenterol.

[22] Peng J, Lu JJ, Zhu J, Xu Y, Lu H, Lian P, Cai G, Cai S (2008). Prediction of treatment outcome by CD44v6 after total mesorectal excision in locally advanced rectal cancer. Cancer J.

[23] Zlobec I, Günthert U, Tornillo L, Iezzi G, Baumhoer D, Terracciano L, Lugli A (2009). Systematic assessment of the prognostic impact of membranous CD44v6 protein expression in colorectal cancer. Histopathology.

[24] Avoranta ST, Korkeila EA, Syrjänen KJ, Pyrhönen SO, Sundström JT (2012). Lack of CD44 variant 6 expression in rectal cancer invasive front associates with early recurrence. World J Gastroenterol.

[25] Rao G, Wang H, Li B, Huang L, Xue D, Wang X, Jin H, Wang J, Zhu Y, Lu Y, Du L, Chen Q (2013). Reciprocal interactions between tumor-associated macrophages and CD44-positive cancer cells via osteopontin/CD44 promote tumorigenicity in colorectal cancer. Clin Cancer Res.

[26] Garouniatis A, Zizi-Sermpetzoglou A, Rizos S, Kostakis A, Nikiteas N, Papavassiliou AG. FAK (2013). CD44v6, c-Met and EGFR in colorectal cancer parameters: tumour progression, metastasis, patient survival and receptor crosstalk. Int J Colorectal Dis.

[27] Li XD, Ji M, Wu J, Jiang JT, Wu CP (2013). Clinical significance of CD44 variants expression in colorectal cancer. Tumori.

[28] Saito S, Okabe H, Watanabe M, Ishimoto T, Iwatsuki M, Baba Y, Tanaka Y, Kurashige J, Miyamoto Y, Baba H (2013). CD44v6 expression is related to mesenchymal phenotype and poor prognosis in patients with colorectal cancer. Oncol Rep.

[29] Lee HJ, Eom DW, Kang GH, Han SH, Cheon GJ, Oh HS, Han KH, Ahn HJ, Jang HJ, Han MS (2013). Colorectal micropapillary carcinomas are associated with poor prognosis and enriched in markers of stem cells. Mod Pathol.

[30] Wang FL, Wan DS, Lu ZH, Fang YJ, Li LR, Chen G, Wu XJ, Ding PR, Kong LH, Lin JZ, Pan ZZ (2013). Expression of molecular markers detected by immunohistochemistry and risk of lymph node metastasis in stage T1 and T2 colorecrectal cancers. Zhonghua Zhong Liu Za Zhi.

[31] Chen L, Jiang B, Wang Z, Liu M, Yang H, Xing J, Zhang C, Yao Z, Zhang N, Cui M, Su X (2014). Combined preoperative CEA and CD44v6 improves prognostic value in patients with stage I and stage II colorectal cancer. Clin Transl Oncol.

[32] Zhao LH, Lin QL, Wei J, Huai YL, Wang KJ, Yan HY (2015). CD44v6 expression in patients with stage II or stage III sporadic colorectal cancer is superior to CD44 expression for predicting progression. Int J Clin Exp Pathol.

[33] Chang JC (2016). Cancer stem cells: Role in tumor growth, recurrence, metastasis, and treatment resistance. Medicine (Baltimore).

[34] Cheng B, Yang G, Jiang R, Cheng Y, Yang H, Pei L, Qiu X (2016). Cancer stem cell markers predict a poor prognosis in renal cell carcinoma: a meta-analysis. Oncotarget.

[35] Todaro M, Gaggianesi M, Catalano V, Benfante A, Iovino F, Biffoni M, Apuzzo T, Sperduti I, Volpe S, Cocorullo G, Gulotta G, Dieli F, De Maria R, Stassi G (2014). CD44v6 is a marker of constitutive and reprogrammed cancer stem cells driving colon cancer metastasis. Cell Stem Cell.

[36] Wang Z, von Au A, Schnölzer M, Hackert T, Zöller M (2016). CD44v6-competent tumor exosomes promote motility, invasion and cancer-initiating cell marker expression. Oncotarget.

[37] Matzke-Ogi A, Jannasch K, Shatirishvili M, Fuchs B, Chiblak S, Morton J, Tawk B, Lindner T, Sansom O, Alves F, Warth A, Schwager C, Mier W (2016). Inhibition of Tumor Growth and Metastasis in Pancreatic Cancer Models by Interference With CD44v6 Signaling. Gastroenterology.

[38] Wang J, Xiao L, Luo CH, Zhou H, Zeng L, Zhong J, Tang Y, Zhao XH, Zhao M, Zhang Y (2015). CD44v6 promotes β-catenin and TGF-β expression, inducing aggression in ovarian cancer cells. Mol Med Rep.

[39] Lv L, Liu HG, Dong SY, Yang F, Wang QX, Guo GL, Pan YF, Zhang XH (2016). Upregulation of CD44v6 contributes to acquired chemoresistance via the modulation of autophagy in colon cancer SW480 cells. Tumour Biol.

[40] Wang L, Liu Q, Lin D, Lai M (2015). CD44v6 down-regulation is an independent prognostic factor for poor outcome of colorectal carcinoma. Int J Clin Exp Pathol.

[41] Afify A, Durbin-Johnson B, Virdi A, Jess H (2016). The expression of CD44v6 in colon: from normal to malignant. Ann Diagn Pathol.

